# A method for identifying neoantigens through isolation of circulating tumor cells using apheresis among patients with advanced-stage cancer

**DOI:** 10.3389/fimmu.2025.1609116

**Published:** 2025-07-11

**Authors:** Daiki Kobayashi, Takuya Kosumi, Queenie Lai Kwan Lam, Shigeharu Fujita, Yasuki Hijikata, Kaori Takeda, Tomoya Narita, Naomi Yamashita, Guilhem Richard, Anne S. De Groot, Naohide Yamashita

**Affiliations:** ^1^ Division of General Internal Medicine, Department of Medicine, Tokyo Medical University Ibaraki Medical Center, Ibaraki, Japan; ^2^ Fujita Health University, Toyoake, Japan; ^3^ Clinic Grandsoul, Nara, Japan; ^4^ Meiko CIT Clinic, Tokyo, Japan; ^5^ Hijikata Clinic, Nagoya, Japan; ^6^ Biomedica Solution Inc., Osaka, Japan; ^7^ Research Institute of Pharmaceutical Sciences, University of Musashino, Tokyo, Japan; ^8^ EpiVax Inc., Providence, RI, United States; ^9^ Novacellum Inc., Tokyo, Japan

**Keywords:** apheresis, CTC, neoantigen, CD45, EpCAM, Vimentin

## Abstract

**Background:**

Immune checkpoint inhibitors show limited efficacy in tumors with low tumor mutational burden, partly due to insufficient neoantigen presentation.

**Methods:**

We developed a novel approach for neoantigen identification using circulating tumor cells (CTCs) isolated via leukapheresis and flow cytometry. Peripheral blood mononuclear cells (PBMCs) were collected from 11 stage IV cancer patients and 2 healthy volunteers. CTCs were enriched by depleting CD45^+^ hematopoietic cells and selecting CD45^−^Vimentin^+^ cells, which were confirmed cytologically to contain malignant cells. Hematopoietic lineage analysis showed that over 50% of the CTC fraction consisted of non-hematopoietic cells. DNA extracted from both the CTC and normal hematopoietic fractions underwent exome sequencing. Neoantigens were identified using the Ancer^®^ bioinformatics platform.

**Results:**

In representative patients with gastric and salivary gland cancers, 94,636 and 46,423 CTCs were isolated, respectively. DNA yields were sufficient for exome sequencing without amplification or extensive cell culture. A total of 102 (patient with gastric cancer) and 108 (patient with salivary gland cancer) neoantigens were identified in each subject, including high-ranking T-cell epitopes derived from single nucleotide variants and frameshift mutations. According to the same procedures we could successfully identify a large number of neoantigens from the CTCs of all stage IV cancer patients. This confirms the feasibility of identifying individual patient-specific neoantigens from CTCs without requiring tumor biopsies.

**Conclusions:**

This is the first study to demonstrate successful neoantigen identification using non-amplified CTCs isolated by apheresis and flow cytometry. The approach provides a minimally invasive, scalable alternative for neoantigen discovery and may better capture tumor heterogeneity compared to single-site biopsies. This method holds promise for enabling rapid, personalized immunotherapy strategies, including peptide vaccines, dendritic cell vaccines, and mRNA-based treatments.

## Introduction

The advent of immune checkpoint inhibitors, first introduced in the early 2000s for metastatic melanoma patients, marked a significant milestone in cancer treatment and brought immunotherapy into the spotlight (1). Although the complete response (CR) rate for immune checkpoint inhibitors remains low, the overall disease control rate (which includes stable disease, SD) has contributed to prolonged overall survival for many patients (2, 3). However, the disease-control rate is still only 50% (4), highlighting the need for new means of improving therapeutic outcomes. Even though response rates have improved since the early days of immune checkpoint inhibitor development, the estimated response rate for ipilimumab—approved for unresectable or metastatic melanoma – has only improved from 0.14% in 2011 to 12.46% in 2018 (5). The response rate of immune checkpoint inhibitors varies significantly depending on the patient’s tumor mutational burden (TMB), cancer type, and concomitant therapy (6). Immunotherapy drugs have faced a major challenge in treating patients with low TMB whose tumors harbor a limited number of potential neoantigens, the targets of T cells unleashed by checkpoint inhibitors, hindering their efficacy (7). To overcome this limitation, additional strategies are needed to enhance the therapeutic effectiveness of immune checkpoint inhibitors.

Neoantigens are tumor-specific antigens produced by tumor cells through mechanisms such as genomic mutations, dysregulated RNA splicing, abnormal post-translational modifications, and viral open reading frames (8). Neoantigens, unlike tumor-associated antigens (TAAs) which are non-mutated self-antigens, are not subject to central T cell tolerance and have been shown to be highly immunogenic (9). Bypassing central tolerance mechanisms enables the immune system to activate stronger tumor-specific immune responses. As a result, neoantigen-based therapies have the potential to significantly improve treatment outcomes by enabling precise targeting of individual tumors. However, many challenges remain to be overcome for unlocking personalized tumor therapies on a large scale. The collection of tumor biopsies, a critical first step in developing personalized therapies, imposes practical and ethical constraints and restricts treatment to patients with resectable tumors. Circulating tumor cells (CTCs), released into the bloodstream, offer a minimally invasive alternative. CTCs have served as biomarkers for early detection, prognosis, and treatment monitoring of cancers (10). Although methods for CTC enrichment via apheresis and flow cytometry have been reported (11–13), the utility for neoantigen identification remains unexplored. Here, we present a novel workflow combining CTC isolation by FACS and exome sequencing to detect tumor-specific neoantigens directly from blood, offering a new direction for personalized immunotherapy.

## Methods

### Patient selection

This study was approved by the institutional review board of Medical Cooperation “Isokai” (approval number: 201903), the nonprofit organization “Kodomotachino Kodomotachino Kodomotachinotameni” (approval number: 21-1), and Musashino University (approval number: R1-1). Peripheral blood mononuclear cells (PBMCs) were obtained from eleven stage IV cancer patients (one with lung cancer, two with pancreatic cancer, one with ampullary cancer, two with breast cancer, one with hepatocellular carcinoma, one with biliary tract cancer, one with rhabdomyosarcoma, one with stomach cancer, and one with salivary gland cancer) after written informed consent was obtained. Patients underwent dendritic cell-based immunotherapy pulsed with tumor-associated antigens at Meiko CIT Clinic or Kyushu Koseikai Clinic. PBMCs were also collected from two healthy volunteers as controls after written informed consent was obtained. Leukapheresis was performed on these volunteers at Meiko CIT Clinic.

### Leukapheresis

PBMCs were obtained from 5-liter leukapheresis procedures performed on patients and healthy volunteers using the COBE SPECTRA (Cobe Laboratories, Lakewood, CO, USA), as described previously (14, 15). The collected cells were then separated by density gradient centrifugation using Ficoll-Hypaque (Pharmacia Biotech, Uppsala, Sweden). The light-density fraction, located at the 42.5–50% interface, was carefully recovered for further processing. The cells were then re-suspended in cold phosphate-buffered saline (PBS) and incubated on 10 cm plastic dishes (Primaria™™, Becton Dickinson, Mountain View, CA, USA) at 37°C for 30 minutes to allow adherence. After incubation, the non-adherent cells (monocyte-depleted PBMCs; m-PBMCs) were collected and cryopreserved in Cell Banker (ZENOAQ, Fukushima, Japan) for circulating tumor cell (CTC) isolation. The remaining adherent cells were used for dendritic cell (DC) preparation.

### Flow cytometric analysis and sorting

After thawing, the cryopreserved cells were washed with PBS containing 2% fetal bovine serum (FBS) and 2 mM ethylenediaminetetraacetic acid (EDTA), then used subsequent staining analysis and cell sorting. Antibodies were added at optimal concentrations, followed by incubation at 4°C for 30 minutes, allowing for staining of the target cell populations. The following antibodies were used in the study: CD45 PE (BioLegend, San Diego, CA, USA), Cell-Surface Vimentin (CSV, clone 84-1) APC (Abnova, Taipei, Taiwan), PE mouse IgG1 κ isotype control and APC mouse IgG2b κ isotype control (BioLegend), Pacific Blue™ anti-human Lineage Cocktail (BioLegend), Human Lineage Cocktail 4 (BD Biosciences Pharmingen), and EpCAM FITC (Biomab, Taipei, Taiwan). To exclude dead cells, propidium iodide (PI; Sigma-Aldrich) staining or 7-Aminoactinomycin D (7AAD) was occasionally used. Cells were analyzed and sorted using an SH800 cell sorter (Sony, Tokyo, Japan).

### Papanicolaou staining

About two to three thousand cells that had been obtained as described above were suspended in a small amount of PBS, smeared onto glass slides, and fixed using M-FIX™ Spray (Sigma-Aldrich). After fixation, the cells were subjected to Papanicolaou staining for cytological analysis.

### Deoxyribonucleic acid extraction

DNA was extracted from the sorted cell fractions using the QIAamp DNA Mini Kit (Qiagen GmbH, Germany) according to the manufacturer’s instructions. The concentration of DNA in all samples was evaluated by Quantus™ Fluorometer (Promega Corporation, Madison, WI, USA) using QuantiFluor^®^ ONE dsDNA System (Promega Corporation). Samples with a DNA concentration of at least 240 ng were used for exome sequencing analysis.

### Exome sequencing

Exome sequencing was outsourced to the Kazusa DNA Research Institute (Chiba, Japan).

#### Exome sequencing conditions

The Twist exome panel was used, covering an approximate region size of 50 Mbp. Library preparation was performed using ultrasonic fragmentation with the Picoruptor and the KAPA Hyper Prep kit.

#### Analysis conditions

The analysis was conducted at the DNA Chip Research Institute. Coverage ranged from approximately 100X to 200X (Seq QC standard), with a target of 10 Gb and surface coverage of 200X. The reference genome used was hg38. Tumor-normal somatic analysis was performed using Strelka, and HLA typing was carried out with Kourami. The custom exome panel covered the combined gene regions of TWIST Biosciences’ TWIST Alliance VCGS Exome and TWIST Exome 2.0. Sequencing was run on Illumina’s NextSeq2000 in 150 base PE mode, generating VCF files.

### Neoantigen identification

VCF files generated from the exome sequencing were analyzed with the Ancer^®^ platform (EpiVax Therapeutics) to identify patient-specific neoantigens (16). Briefly, paired normal/mutated amino acid segments were extracted for each mutation yielding non-synonymous changes. Paired sequences were then assessed by EpiMatrix for the presence of putative HLA Class I and HLA Class II T-cell epitopes restricted by the patients’ HLAs. Predicted T-cell epitopes that were newly detected in tumor sequences or that exhibited a significant change in EpiMatrix score, as compared to their normal counterpart, were labeled as neoepitopes. Neoepitopes were further screened with JanusMatrix to flag and remove those sequences with extensive cross-conservation with the human proteome. Source mutated sequences were then trimmed to generate 14- to 25-mer neoantigen sequences containing non-self HLA Class I and/or HLA Class II neoepitopes. Neoantigens were subsequently ranked based on their predicted immunogenicity and assigned an Ancer score.

## Results

### Flow cytometric analysis of m-PBMCs

**Figure 1:**
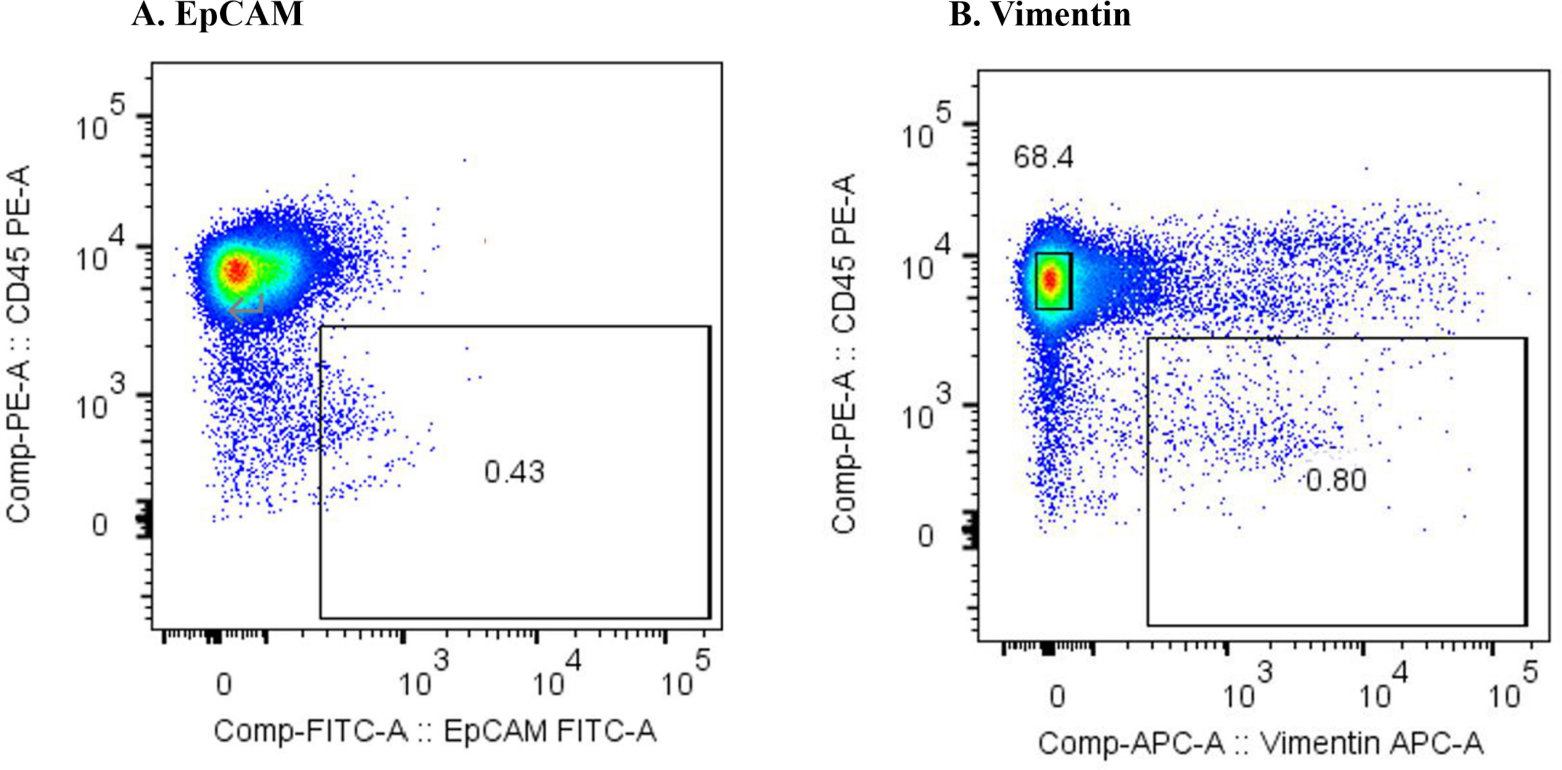
**(A)** Flow cytometric analysis of monocyte-depleted PBMCs(m-PBMCs) obtained from an ampullary cancer patient. The y-axis indicates the intensity of CD45 PE and the x-axis, that of EpCAM FITC. **(B)** Flow cytometric analysis of m-PBMC obtained from the same patient. The y-axis indicates the intensity of CD45 PE and the x-axis, that of vimentin APC.

**Figure 2:**
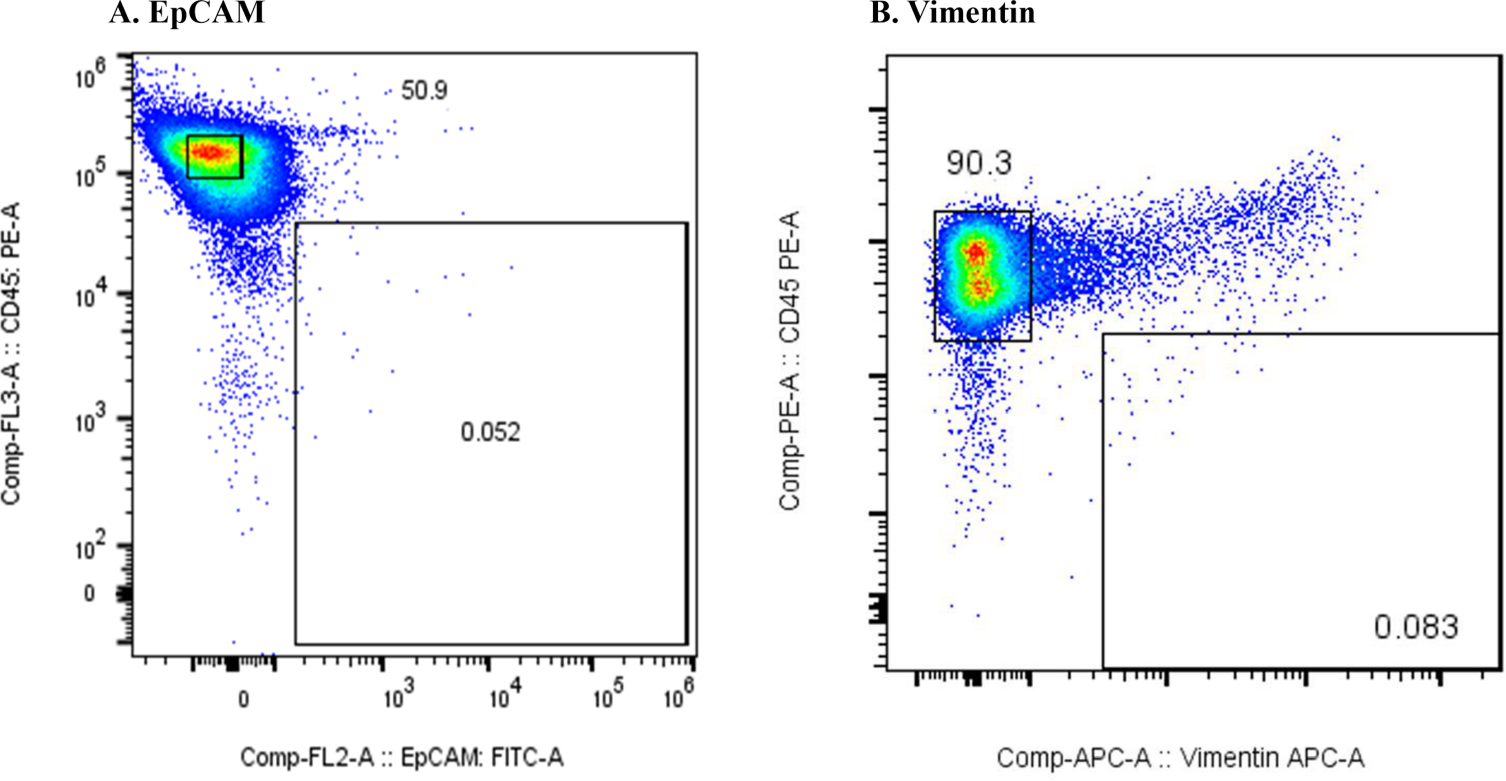
**(A)** Flow cytometric analysis of monocyte-depleted PBMCs(m-PBMCs) obtained from a healthy volunteer. The y-axis indicates the intensity of CD45 PE and the x-axis, that of EpCAM FITC. **(B)** Flow cytometric analysis of m-PBMC obtained from the same subject. The y-axis indicates the intensity of CD45 PE and the x-axis, that of vimentin APC.

**Figure 1** shows the results of the flow cytometric analysis of m-PBMCs from a stage IV patient with ampullary cancer, stained with anti-EpCAM antibody (**Figure 1A**) and anti-Vimentin antibody (**Figure 1B**). The proportions of CD45^−^EpCAM^+^ and CD45^−^Vimentin^+^ cells were 0.43% and 0.80%, respectively. The intensity of CD45^−^EpCAM^+^ cells staining was considerably lower than CD45^−^Vimentin^+^ cells, making it difficult to conclusively determine the presence of CD45^−^EpCAM^+^ cells in **Figure 1A**. The proportion of CD45^−^EpCAM^+^ cells and CD45^−^Vimentin^+^ cells were found to be very low in samples collected from two healthy volunteers, with values of 0.057% and 0.083%, respectively, observed for the first volunteer (**Figures 2A, B**), and 0.072% and 0.057%, respectively, for the second volunteer.

**Figure 3:**
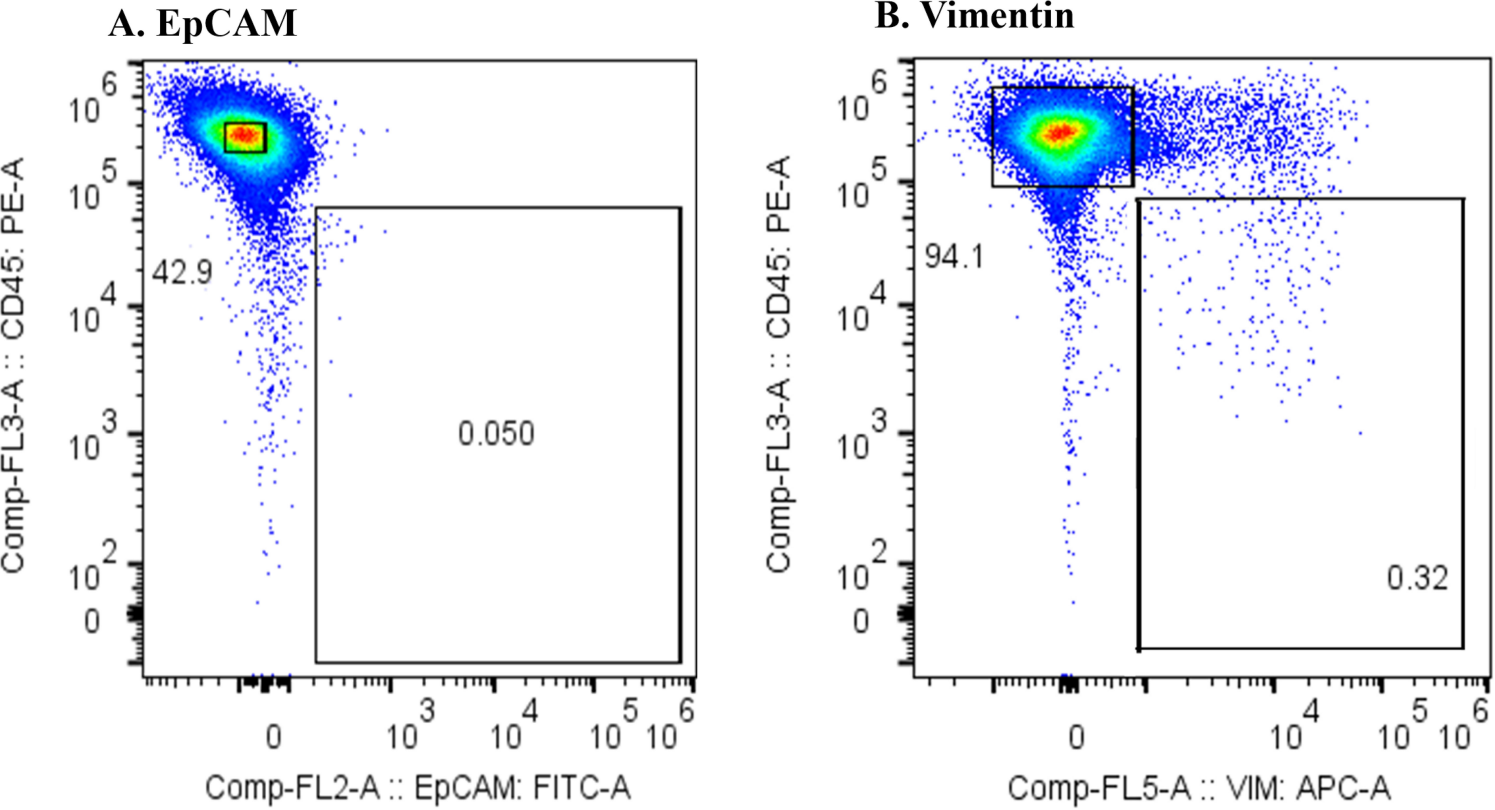
**(A)** Flow cytometric analysis of monocyte-depleted PBMCs(m-PBMCs) obtained from a pancreatic cancer patient. The y-axis indicates the intensity of CD45 PE and the x-axis, that of EpCAM FITC. **(B)** Flow cytometric analysis of m-PBMC obtained from the same patient. The y-axis indicates the intensity of CD45 PE and the x-axis, that of vimentin APC.

**Figure 3** presents the results of the flow cytometric analysis of m-PBMCs from a stage IV pancreatic cancer patient. The CD45^−^EpCAM^+^ fraction (**Figure 3A**) contained very few cells (0.05%), whereas the CD45^−^Vimentin^+^ fraction (**Figure 3B**) had a higher cell count at 0.32%. CD45^−^Vimentin^+^ cell staining exhibited higher intensity than CD45^−^EpCAM^+^ cells, a pattern that was consistent in m-PBMCs across all stage IV cancer patients analyzed in this study.

Epithelial malignant tumor cells typically express the epithelial marker EpCAM on their surface; however, they often undergo epithelial-mesenchymal transition (EMT), leading to the loss of EpCAM and the expression of mesenchymal markers such as vimentin. EMT is known to occur frequently in pancreatic cancer (17), which aligns with the results in **Figure 3**. It is likely that CTCs from the cancer patients included in this study had undergone substantial EMT, reducing the number of cells detected with the anti-EpCAM antibody. Furthermore, reports suggest that CTCs are more effectively captured by the anti-Vimentin antibody than by the anti-EpCAM antibody (18). Therefore, the subsequent study was conducted using the anti-Vimentin antibody. In the flow cytometric analysis of m-PBMCs obtained from all stage IV cancer patients via apheresis, a significant number of cells were observed in the CD45^−^Vimentin^+^ fractions, whereas these fractions contained very few cells in healthy individuals. This suggested that the cells in the CD45^−^Vimentin^+^ fractions may represent circulating tumor cells (CTCs). To confirm this hypothesis, the following experiments were conducted.

### Cytology and hematopoietic lineage investigation of CD45^-^Vimentin^+^ fraction cells

**Figure 4:**
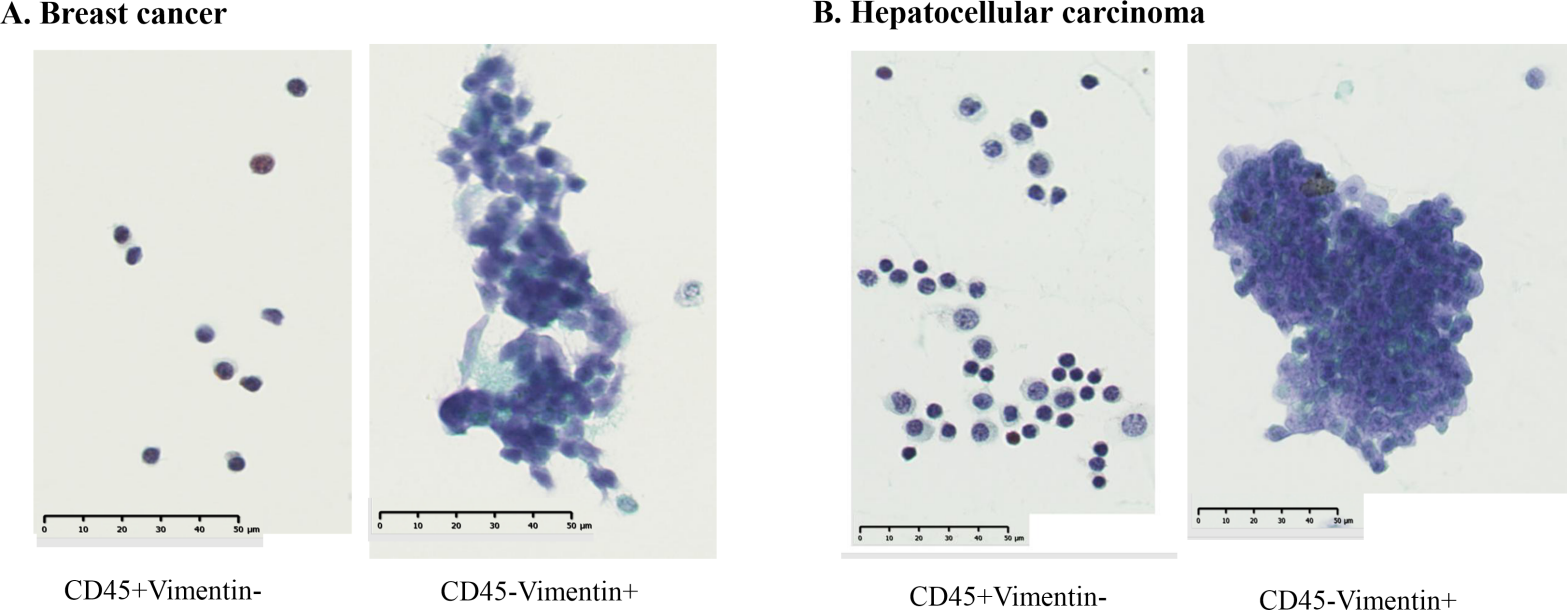
Cytological examination of CD45^+^Vimentin^-^ and CD45^-^Vimentin^+^ fraction cells. Papanicolaou staining was done on smeared and fixed cells. **(A)** Cells of CD45^+^Vimentin^-^ and CD45^-^Vimentin^+^ fraction obtained from a stage IV breast cancer patient. **(B)** Cells of CD45^+^Vimentin^-^ and CD45^-^Vimentin^+^ fraction obtained from a stage IV breast hepatocellular carcinoma patient.

Next, we examined whether cancer cells were present in the CD45^-^Vimentin^+^ fraction by cytological examination. After sorting the cells in the CD45^+^Vimentin^-^ and CD45^-^Vimentin^+^ fractions, smeared and fixed cells were stained with Papanicolaou stain, as shown in **Figure 4**. **Figure 4A** shows the results from a stage IV breast cancer patient: all cells in the CD45^+^Vimentin^-^ fraction were normal lymphocytes. In contrast, the CD45^-^Vimentin^+^ fraction contained a cluster of atypical cells, with a pathological diagnosis of Class V. **Figure 4B** shows cells from a stage IV hepatocellular carcinoma patient. Similar to **Figure 4A**, only normal lymphocytes (small and large lymphocytes) were observed in the CD45^+^Vimentin^-^ fraction. In the CD45^-^Vimentin^+^ fraction, however, a cluster of atypical cells was also seen, with again a pathological diagnosis of Class V. Based on these results, the CD45^-^Vimentin^+^ fraction, which we presumed to be the CTC fraction, indeed contains cancer cells.

**Figure 5:**
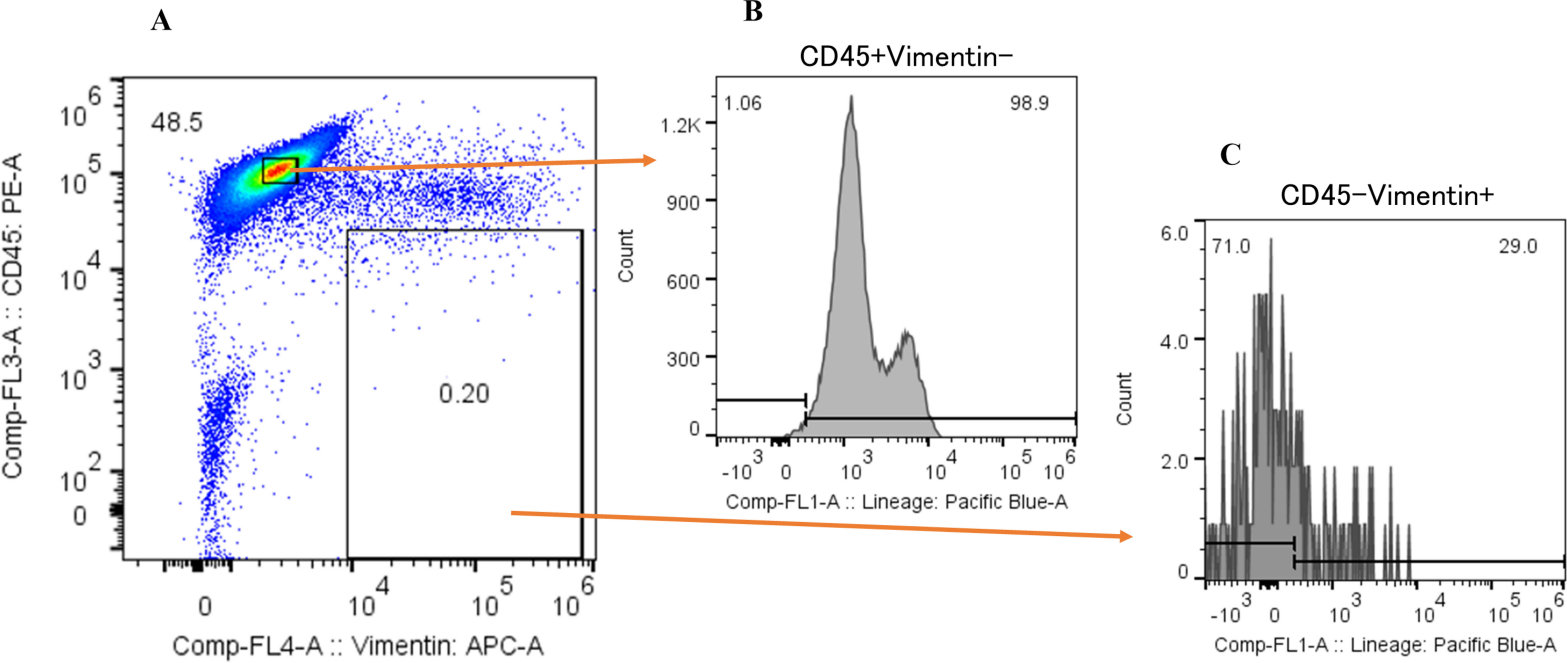
Hematopoietic lineage investigation of CD45^-^Vimentin^+^ fraction cells. **(A)** Flow cytometric analysis of m-PBMC obtained from a hepatocellular carcinoma patient. The y-axis indicates the intensity of CD45 PE and the x-axis, that of vimentin APC. **(B)** Flow cytometric lineage analysis of CD45^+^Vimentin^-^ fraction cells in **(A)**. The y-axis indicates the event of cells and the x-axis, the intensity of human lineage cocktail 4 Pacific blue. **(C)** Flow cytometric lineage analysis of CD45^-^Vimentin^+^ fraction cells in **(A)**. The y-axis indicates the event of cells and the x-axis, the intensity of human lineage cocktail 4 Pacific blue.

We next examined the proportion of cancer cells within the CD45^-^Vimentin^+^ fraction (CTC fraction). In this analysis, we used the Human Lineage Cocktail 4 (containing antibodies against CD2, CD3, CD4, CD7, CD8, CD10, CD11b, CD14, CD19, CD20, CD56, and CD235a), which reacts with most hematopoietic cells, to assess the extent of hematopoietic cell contamination in the CTC fraction. This would enable the estimation of the proportion of cancer cells within this fraction. **Figure 5** shows the lineage analysis of hematopoietic cells in the CTC fraction of a hepatocellular carcinoma patient. The CTC fraction of this patient (**Figure 5A**) was reanalyzed with anti-CD45 antibody and anti-human Lineage Cocktail 4, revealing that 71.0% of the cells were non-hematopoietic (**Figure 5B**: CD45^+^Vimentin^-^, **Figure 5C**: CD45^-^Vimentin^+^), suggesting that these cells are circulating cancer cells. **Table 1** presents the results of hematopoietic lineage analysis in the m-PBMCs of four additional patients, showing that 50.2% to 73.4% of the cells in the CTC fraction could be categorized as cancer cells. In summary, these results confirm the presence of cancer cells in the CTC fraction, with a purity of at least 50%.

**Table 1:** Hematopoietic lineage analysis in the m-PBMCs obtained from stage IV cancer patients.

Cancer Type	lineage-(%)	lineage+(%)
hepatocellular carcinoma	71.0	29.0
rhabdomyosarcoma	50.2	49.8
biliary tract canccer	73.4	26.6
breast cancer 1	71.2	28.8
breast cancer 2	72.4	27.8

### Identification of neoantigens using cells from the CTC fraction and normal hematopoietic cells

**Figure 6:**
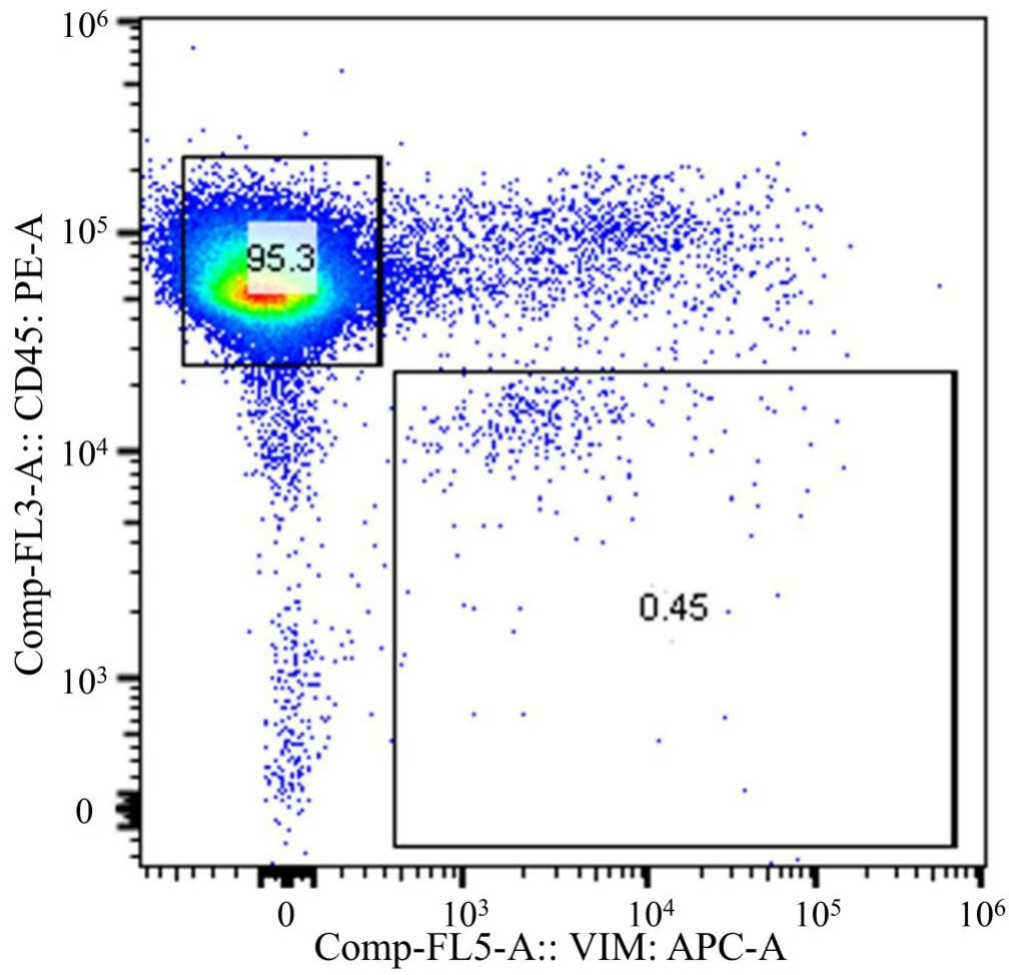
Flow cytometric analysis of monocyte-depleted PBMCs(m-PBMCs) obtained from a stage IV gastric cancer patient. The y-axis indicates the intensity of CD45 PE and the x-axis, that of vimentin APC.

To achieve accurate neoantigen identification, exome sequencing must be performed by amplification of DNA or culturing of CTCs obtained from the circulating tumor cells. This requires both high purity of the cell population, and a sufficient quantity of CTCs. The CTC fraction sorted in this study met these conditions without nucleic acid amplification or cell culture, allowing us to proceed with exome sequencing and neoantigen identification. **Figure 6** shows the flow cytometric analysis of m-PBMCs from a stage IV gastric cancer patient. The m-PBMCs from this patient were divided into four vials, with the analysis of one vial shown in **Figure 6**. Sorting was performed to separate the CD45^+^Vimentin^-^ fraction (normal hematopoietic fraction), consisting of normal hematopoietic cells, and the CD45^-^Vimentin^+^ fraction (CTC fraction), mainly composed of CTCs. A total of 45,665 cells were obtained from the CTC fraction. Sorting of the normal hematopoietic fraction was stopped once 3 million cells were collected. Another vial of m-PBMCs was similarly sorted, yielding 48,971 cells from the CTC fraction. In total, 94,636 cells were collected from the CTC fraction contained in two vials of m-PBMCs, and 360 ng of DNA was extracted from these isolated CTC. Next, 1,260 ng of DNA was extracted from the 3 million cells in the normal hematopoietic fraction.

**Figure 7:**
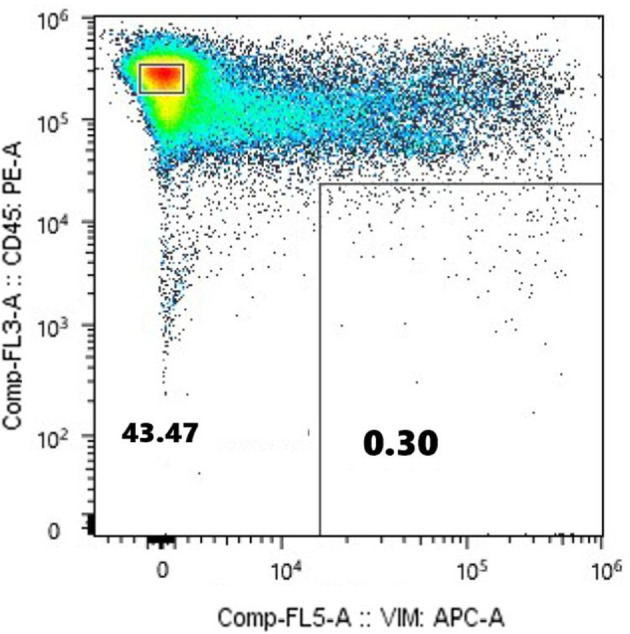
Flow cytometric analysis of monocyte-depleted PBMCs(m-PBMCs) obtained from a stage IV salivary gland cancer patient. The y-axis indicates the intensity of CD45 PE and the x-axis, that of vimentin APC.

Exome sequencing was performed on DNA obtained from the normal hematopoietic fraction and the CTC fraction, followed by neoantigen identification. Exome sequencing of the CD45^+^Vimentin^-^ cells (normal hematopoietic fraction) and the CD45^-^Vimentin^+^ cells (CTC fraction) were carried out, and 102 neoantigens were identified using the Ancer platform in the patient with gastric cancer. The top ranked 30 neoantigens identified with Ancer are shown in **Table 2**, where long neoantigen peptides may contain multiple overlapping (short) HLA-binding cores. Most neoantigens were derived from single nucleotide variations (SNVs), although one neoantigen (ranked #1) was identified from a frameshift mutation. **Figure 7** shows the flow cytometric profile of a stage IV salivary gland cancer patient, from which CTC were collected. A total of 603 ng of DNA was extracted from the 46,423 cells in the CTC fraction of this patient. Exome sequencing of the CD45^+^Vimentin^-^ cells (normal hematopoietic fraction) and the CD45^-^Vimentin^+^ cells (CTC fraction) were carried out, and 108 neoantigens were identified using the Ancer platform in the patient with salivary gland cancer. The top ranked 30 neoantigens are shown in **Table 3** similar to **Table 2**. A neoantigen derived from frameshift mutation (ranked #21) was also detected. According to the above procedures we could successfully identify a large number of neoantigens from the CTCs of all stage IV cancer patients examined to date.

**Table 2:** Neoantigens identified in a stage IV gastric cancer patient.

Rank	Gene	AA Mutation	Sequence ID	Optimized Mutated Sequence	Immunogenicity Score		VAF	Ancer^®^ Score	Minimal Neoepitopes: HLA Restriction
					Class I	Class II			
1	ZNF717	Thr577ProfsTer51	ZNF717_ENST00000478296_THR577PROFSTER51	IRELTQERNLTYVMNVEKPFIASHS	13.04	2.7	0.158	2.485	LTQERNLTY: DRB1*0101, LTQERNLTY: B1501 (strong ligand), TYVMNVEKPF: A2402 (strong ligand), YVMNVEKPF: A2402, YVMNVEKPF: B0702, YVMNVEKPF: B1501 (strong ligand), YVMNVEKPFI: B1501, VMNVEKPFI: DRB1*1501
2	KLK10	Ala34Val	KLK10_ENST00000309958_ALA34VAL	AQLWAAEAVLLPQNDTR	5.71	4.44	0.046	0.470	AQLWAAEAVL: B1501, LWAAEAVLL: DRB1*0101, LWAAEAVLL: DRB1*1501, LWAAEAVLL: A2402, LWAAEAVLLP: A2402
3	FZR1	Thr298Met	FZR1_ENST00000313639_THR298MET	IRFWNMLTGQPLQC	7.65	3.08	0.042	0.447	FWNMLTGQPL: A2402 (strong ligand), FWNMLTGQPL: B0702, WNMLTGQPL: DRB1*0101 (strong ligand), WNMLTGQPL: A2402
4	ZNF778	Lys122Thr	ZNF778_ENST00000306502_LYS122THR	IPCQKTLFTIGEQFSVL	15.06	0	0.028	0.418	IPCQKTLFTI: A2402, IPCQKTLFTI: B0702 (strong ligand), PCQKTLFTI: A2402, KTLFTIGEQF: B1501, TLFTIGEQF: A2402, TLFTIGEQF: B1501 (strong ligand), FTIGEQFSVL: B1501
5	LEMD3	Ser164Pro	LEMD3_ENST00000308330_SER164PRO	RDQAGGGGRKDRAPLQYRGLKAPP	5.17	0	0.069	0.355	GGRKDRAPL: B0702 (strong ligand), APLQYRGLK: B0702, APLQYRGLKA: B0702
6	PI4KB	Arg542Trp	PI4KB_ENST00000368872_ARG542TRP	REGSPYGHLPNWWLLSVIVKC	20.18	3.82	0.014	0.347	SPYGHLPNWW: A2402, PYGHLPNWWL: A2402 (strong ligand) YGHLPNWWL: DRB1*0101, GHLPNWWLL: A2402, LPNWWLLSV: B0702 (strong ligand), LPNWWLLSVI: A2402, LPNWWLLSVI: B0702 (strong ligand), WWLLSVIVK: DRB1*0101, WWLLSVIVKC: A2402
7	MAML3	Leu77Pro	MAML3_ENST00000509479_LEU77PRO	KHSTVVERPRQRIEGCRRHHVNCE	3.92	0	0.087	0.339	VVERPRQRI: B0702, RPRQRIEGC: B0702
8	ME3	Arg435His	ME3_ENST00000393324_ARG435HIS	RDMASFHEHPIIFALS	9.83	0	0.034	0.333	RDMASFHEHP: A2402, MASFHEHPI: B0702, MASFHEHPII: A2402, ASFHEHPIIF: B1501, SFHEHPIIF: A2402
9	BHLHE41	Ser147Ala	BHLHE41_ENST00000242728_SER147ALA	TCAKEVLQYLARFESWTPREPRC	7.6	3.86	0.028	0.318	LQYLARFES: DRB1*0101, LQYLARFES: DRB1*1501, LQYLARFESW: A2402, QYLARFESW: A2402 (strong ligand), QYLARFESWT: A2402
10	NUP210	Ala1540Thr	NUP210_ENST00000254508_ALA1540THR	GSVTVYYEVTGHLRT	8.39	1.77	0.028	0.282	TVYYEVTGHL: A2402, TVYYEVTGHL: B1501, VYYEVTGHL: A2402 (strong ligand), VYYEVTGHLR: A2402 (strong ligand), YYEVTGHLR: DRB1*0101
11	IGF2R	Asn2020Ser	IGF2R_ENST00000356956_ASN2020SER	SLVHSGVSYYINLC	8.69	4.13	0.022	0.279	SLVHSGVSY: B1501 (strong ligand), SLVHSGVSYY: B1501 (strong ligand), LVHSGVSYY: B1501 (strong ligand), VHSGVSYYI: DRB1*0101, VHSGVSYYI: DRB1*1501 (strong ligand)
12	SAP18	Gly57Ala	SAP18_ENST00000382533_GLY57ALA	RVFTTNNARHHRMDEFSRGNVPS	6.42	1.64	0.031	0.248	RVFTTNNAR: B1501, RVFTTNNARH: B1501 (strong ligand), TTNNARHHRM: B1501
13	KCNN3	Ala110Gly	KCNN3_ENST00000271915_ALA110GLY	HSSPTAFRGPPSSNST	4.38	1.87	0.038	0.238	SPTAFRGPP: B0702, SPTAFRGPPS: B0702, FRGPPSSNS: DRB1*1501
14	IRAK1	Phe196Ser	IRAK1_ENST00000369974_PHE196SER	ESSVSLLQGARPSPFCWP	10.77	0	0.022	0.237	LLQGARPSPF: A2402, LLQGARPSPF: B1501 (strong ligand), LQGARPSPF: A2402, LQGARPSPF: B1501 (strong ligand), GARPSPFCW: A2402
15	AATK	Lys181Gln	AATK_ENST00000326724_LYS181GLN	RALQHSNLLQCLAQCAEVT	4.27	3.84	0.025	0.204	RALQHSNLL: DRB1*1501, RALQHSNLL: A2402, RALQHSNLL: B0702 (strong ligand), LQHSNLLQC: DRB1*1501, LQHSNLLQCL: B1501
16	CSNK2A3	Ile133Thr	CSNK2A3_ENST00000528848_ILE133THR	QTLTDYDTRFYMYEILK	10.3	0	0.018	0.188	TLTDYDTRF: B1501, TLTDYDTRFY: B1501, LTDYDTRFY: B1501, TDYDTRFYMY: A2402, TDYDTRFYMY: B1501, DYDTRFYMY: A2402
17	PLA2G4D	Ser434Thr	PLA2G4D_ENST00000290472_SER434THR	VDLWALVLETMLHGQV	3.87	1.68	0.033	0.183	LWALVLETM: A2402 (strong ligand), LWALVLETML: A2402 (strong ligand), LETMLHGQV: DRB1*1501
18	BCL11B	Cys788Tyr	BCL11B_ENST00000345514_CYS788TYR	KTHGQIGKEVYRCDIYQMPFSV	6.34	0	0.028	0.180	VYRCDIYQM: A2402 (strong ligand), YRCDIYQMPF: A2402, YRCDIYQMPF: B1501
19	FAT3	Leu1918Gln	FAT3_ENST00000409404_LEU1918GLN	LKVSATDPDSEVPPEQTYSLMEGS	5.24	0	0.034	0.176	VPPEQTYSL: B0702 (strong ligand), VPPEQTYSLM: B0702 (strong ligand)
20	BIN1	Glu347Asp	BIN1_ENST00000259238_GLU347ASP	EQILSLFDDTFVPE	5.79	0	0.030	0.172	QILSLFDDTF: A2402, ILSLFDDTF: A2402, ILSLFDDTF: B1501
21	RNF43	Arg113Gln	RNF43_ENST00000407977_ARG113GLN	ISIVKLESPQRAPRP	0	5.13	0.033	0.171	IVKLESPQR: DRB1*0101, IVKLESPQR: DRB1*1501, VKLESPQRA: DRB1*0101
22	PAQR6	Glu263Lys	PAQR6_ENST00000335852_GLU263LYS	AHWRGVPRPNNSKAPSLT	5.99	0	0.028	0.165	VPRPNNSKA: B0702 (strong ligand), RPNNSKAPS: B0702 (strong ligand), RPNNSKAPSL: B0702 (strong ligand)
23	INTS9	Gln472His	INTS9_ENST00000416984_GLN472HIS	QSHRMDLMIDCHPPAMSYRRAE	10.08	0	0.016	0.161	LMIDCHPPA: B0702, LMIDCHPPA: B1501, LMIDCHPPAM: B1501 (strong ligand), MIDCHPPAM: B0702, MIDCHPPAM: B1501
24	PTPN23	Glu603Lys	PTPN23_ENST00000265562_GLU603LYS	VTTDHSEMKKLFKEQLKKYDQLKV	4.3	1.7	0.027	0.160	EMKKLFKEQL: A2402, MKKLFKEQL: DRB1*1501, KLFKEQLKKY: B1501
25	OR52E6	Ser95Pro	OR52E6_ENST00000329322_SER95PRO	WFNIKEIPFGGYLSQ	6.93	0	0.022	0.154	WFNIKEIPF: A2402, NIKEIPFGGY: B1501, KEIPFGGYL: A2402, IPFGGYLSQ: B0702
26	TMEM204	Asp130Asn	TMEM204_ENST00000253934_ASP130ASN	GLVGLPLLSPNAPCWEEAM	5.05	0	0.028	0.141	LPLLSPNAP: B0702, LPLLSPNAPC: B0702, LLSPNAPCW: A2402
27	KDM7A	Arg644Ser	KDM7A_ENST00000397560_ARG644SER	KPLNGFFTSVKSEL	7.03	1.66	0.016	0.136	KPLNGFFTSV: B0702 (strong ligand), GFFTSVKSEL: A2402 (strong ligand), FFTSVKSEL: A2402
28	MRGPRF	Lys16Arg	MRGPRF_ENST00000320913_LYS16ARG	MAGNCSWEAHPGNRNRVSATGGGP	3.71	0	0.036	0.133	HPGNRNRVS: B0702, HPGNRNRVSA: B0702 (strong ligand)
29	PTPRQ	Val687Asp	PTPRQ_ENST00000616559_VAL687ASP	RVAASTHDGESSLSEENDIFVRT	3.94	0	0.033	0.131	ASTHDGESSL: B0702, STHDGESSL: B1501
30	DKK2	Lys202Gln	DKK2_ENST00000285311_LYS202GLN	CCARHFWTQICKPVLHQGE	6.78	1.84	0.015	0.130	CARHFWTQI: B0702, FWTQICKPVL: A2402 (strong ligand), FWTQICKPVL: B0702, WTQICKPVL: DRB1*0101

Underlined optimized mutated sequence (Rank 1) indicates the frameshift mutation. AA Mutation: amino acid mutation, Optimized Mutated Sequence: long peptide neoepitope containing multiple shorter T-cell epitopes, Immunogenicity Score: the immunogenic potential of the encoded neoepitopes adjusted for homology with the Human proteome (Higher scores are indicative of a higher immunogenic potential.), VAF: variant allele frequency, Ancer^®^ Score: the parameter determined by EpiVax Therapeutics ranking the neoepitopes, which is based both on how mutations impact HLA- and TCR-interacting residues of predicted T-cell epitopes and on how high the immunogenic potential is.

**Table 3:** Neoantigens identified in a stage IV salivary gland cancer patient.

Rank	Gene	AA Mutation	Sequence ID	Optimized Mutated Sequence	Immunogenicity Score		VAF	Ancer^®^ Score	Minimal Neoepitopes: HLA Restriction
					Class I	Class II			
1	DISP2	Trp168Cys	DISP2_ENST00000267889_TRP168CYS	KSYSQLIAECPVAVLML	30.12	0	0.033	0.980	YSQLIAECPV: A0201, YSQLIAECPV: A0207, YSQLIAECPV: B5101 (strong ligand), SQLIAECPV: A0201, SQLIAECPV: A0207, QLIAECPVA: A0201, QLIAECPVA: A0207, QLIAECPVAV: A0201 (strong ligand), QLIAECPVAV: A0207 (strong ligand), LIAECPVAV: A0201 (strong ligand), LIAECPVAV: A0207 (strong ligand), LIAECPVAV: B5101, LIAECPVAVL: A0201, LIAECPVAVL: A0207, IAECPVAVL: B5101
2	BTBD17	Trp321Cys	BTBD17_ENST00000375366_TRP321CYS	RNYLAPACGAPWVINNP	12.14	0	0.060	0.731	LAPACGAPW: B5101 (strong ligand), LAPACGAPWV: B5101, APACGAPWV: B5101 (strong ligand), APACGAPWVI: A0201, APACGAPWVI: A0207, APACGAPWVI: B5101 (strong ligand)
3	MOB3C	Gly179Ser	MOB3C_ENST00000271139_GLY179SER	LLMDWIESLINDEEVFPTRVGVP	20.46	1.72	0.029	0.634	LLMDWIESL: A0201 (strong ligand), LLMDWIESL: A0207 (strong ligand), LLMDWIESLI: A0201 (strong ligand), LLMDWIESLI: A0207 (strong ligand), LLMDWIESLI: B5101 (strong ligand), LMDWIESLI: A0201, LMDWIESLI: A0207, MDWIESLIN: DRB1*0403
4	KCNH4	Ile379Val	KCNH4_ENST00000264661_ILE379VAL	AHWMACVWYVIGRRE	18.68	0	0.031	0.581	AHWMACVWYV: A0201, AHWMACVWYV: A0207, HWMACVWYV: A0201 (strong ligand), HWMACVWYV: A0207 (strong ligand), HWMACVWYVI: B5101, WMACVWYVI: A0201 (strong ligand), WMACVWYVI: A0207 (strong ligand), WMACVWYVI: B5101
5	PIM1	Val197Leu	PIM1_ENST00000373509_VAL197LEU	DFGSGALLKDTLYTDFDGTR	17.09	0	0.034	0.579	ALLKDTLYT: A0201, ALLKDTLYT: A0207, ALLKDTLYTD: A0201, ALLKDTLYTD: A0207, LLKDTLYTD: A0201, LLKDTLYTD: A0207, TLYTDFDGTR: A0201, TLYTDFDGTR: A0207
6	HMCN2	Arg4966Gln	HMCN2_ENST00000624552_ARG4966GLN	CSQDCGTGGPSTLQYQLLPLPL	11.35	0	0.048	0.540	GPSTLQYQL: B5101, GPSTLQYQLL: B5101, TLQYQLLPL: A0201, TLQYQLLPL: A0207, LQYQLLPLPL: A0201, LQYQLLPLPL: A0207, LQYQLLPLPL: B5101
7	SGTA	Gln13His	SGTA_ENST00000221566_GLN13HIS	MDNKKRLAYAIIHFLHDQLRH	14.05	4.61	0.027	0.507	RLAYAIIHFL: A0201 (strong ligand), RLAYAIIHFL: A0207 (strong ligand), LAYAIIHFL: A0201 (strong ligand), LAYAIIHFL: A0207 (strong ligand), LAYAIIHFL: B5101 (strong ligand), YAIIHFLHD: B5101, IHFLHDQLR: DRB1*0403, IHFLHDQLR: DRB1*0803
8	PLA2G6	Ala333Val	PLA2G6_ENST00000332509_ALA333VAL	NTALHVAVMRNRFDCVIVLLTHGA	13.14	3.83	0.029	0.499	AVMRNRFDCV: A0201, AVMRNRFDCV: A0207, VMRNRFDCVI: A0201, VMRNRFDCVI: A0207, MRNRFDCVI: B5101, FDCVIVLLT: DRB1*0403, VIVLLTHGA: DRB1*0403
9	IGF2	Met9Val	IGF2_ENST00000381389_MET9VAL	MGIPMGKSVLVLLTF	16.86	0	0.030	0.498	MGIPMGKSVL: B5101, GIPMGKSVLV: A0201, GIPMGKSVLV: A0207, IPMGKSVLV: B5101 (strong ligand), IPMGKSVLVL: A0201, IPMGKSVLVL: A0207, IPMGKSVLVL: B5101 (strong ligand)
10	NXF3	Tyr443Phe	NXF3_ENST00000395065_TYR443PHE	HDLSSFLVDMWFQTEWMLC	17.35	0	0.028	0.484	FLVDMWFQT: A0201 (strong ligand), FLVDMWFQT: A0207 (strong ligand), FLVDMWFQTE: A0201, FLVDMWFQTE: A0207, VDMWFQTEW: B5101, VDMWFQTEWM: B5101, DMWFQTEWML: A0201, DMWFQTEWML: A0207
11	EFR3B	Ala229Val	EFR3B_ENST00000264719_ALA229VAL	RQLRLSIDYVLTGSYDGA	15.27	1.06	0.030	0.482	QLRLSIDYV: A0201, QLRLSIDYV: A0207, QLRLSIDYVL: A0201, QLRLSIDYVL: A0207, QLRLSIDYVL: B5101, LRLSIDYVL: B5101, YVLTGSYDG: DRB1*0803
12	PHC2	Met838Ile	PHC2_ENST00000257118_MET838ILE	LKEDHLMSAINIKL	10.22	4.06	0.032	0.464	HLMSAINIKL: A0201 (strong ligand), HLMSAINIKL: A0207 (strong ligand), LMSAINIKL: DRB1*0403, LMSAINIKL: DRB1*0803, LMSAINIKL: A0201 (strong ligand), LMSAINIKL: A0207 (strong ligand)
13	BTBD17	Glu186Gln	BTBD17_ENST00000375366_GLU186GLN	HYAVGTGDEALRQSCLQFLAWN	7.61	0	0.061	0.463	ALRQSCLQFL: A0201, ALRQSCLQFL: A0207, RQSCLQFLA: A0201, RQSCLQFLA: A0207
14	CUEDC1	Lys55Thr	CUEDC1_ENST00000360238_LYS55THR	EFNQAMDDFTTMFPNMD	10.42	2.36	0.036	0.454	QAMDDFTTM: B5101 (strong ligand), AMDDFTTMF: A0201, AMDDFTTMF: A0207, AMDDFTTMFP: A0201, AMDDFTTMFP: A0207, FTTMFPNMD: DRB1*0803 (strong ligand)
15	PHC2	Arg647Trp	PHC2_ENST00000257118_ARG647TRP	KLKCELCGWVDFAYKFKRS	11.55	0	0.039	0.449	KLKCELCGWV: A0201, KLKCELCGWV: A0207, ELCGWVDFA: A0201, ELCGWVDFA: A0207, ELCGWVDFAY: A0201, ELCGWVDFAY: A0207
16	BTBD11	His353Tyr	BTBD11_ENST00000280758_HIS353TYR	KFTVETLEYTVNNDSEIWG	9.58	2.1	0.037	0.430	FTVETLEYT: A0201, FTVETLEYT: A0207, FTVETLEYTV: A0201, FTVETLEYTV: A0207, LEYTVNNDS: DRB1*0403, YTVNNDSEI: A0201, YTVNNDSEI: A0207
17	OR10H4	Pro139Thr	OR10H4_ENST00000322107_PRO139THR	RYNVLMSTRDCAHLVACT	11.21	2.43	0.031	0.427	YNVLMSTRD: DRB1*0803 (strong ligand), VLMSTRDCA: A0201, VLMSTRDCA: A0207, VLMSTRDCAH: B5101, LMSTRDCAHL: A0201, LMSTRDCAHL: A0207, STRDCAHLV: A0201, STRDCAHLV: A0207
18	ADGRD2	Thr701Ser	ADGRD2_ENST00000334810_THR701SER	GCGVSFCALSTTFLLF	16.82	3.8	0.021	0.425	VSFCALSTTF: B5101 (strong ligand), SFCALSTTFL: A0201, SFCALSTTFL: A0207, FCALSTTFL: DRB1*0403, FCALSTTFL: DRB1*0803, FCALSTTFL: A0201, FCALSTTFL: A0207, FCALSTTFLL: A0201, FCALSTTFLL: A0207, CALSTTFLL: A0201, CALSTTFLL: A0207, CALSTTFLL: B5101 (strong ligand)
19	CFAP65	Gln957Arg	CFAP65_ENST00000341552_GLN957ARG	LEETKYLFRVGMWVWE	13.06	0	0.031	0.408	YLFRVGMWV: A0201 (strong ligand), YLFRVGMWV: A0207 (strong ligand), YLFRVGMWV: B5101, YLFRVGMWVW: A0201, YLFRVGMWVW: A0207
20	TMEM82	Gly273Cys	TMEM82_ENST00000375782_GLY273CYS	QSQVQTVLVRMCGLFV	9.27	2.52	0.034	0.400	VLVRMCGLFV: A0201 (strong ligand), VLVRMCGLFV: A0207 (strong ligand), LVRMCGLFV: DRB1*0403 (strong ligand), LVRMCGLFV: A0201, LVRMCGLFV: A0207
21	GPR182	His293Tyr	GPR182_ENST00000300098_HIS293TYR	HGTHISLHCYLVHLLYF	22.51	0	0.018	0.395	GTHISLHCYL: B5101, THISLHCYLV: B5101, HISLHCYLV: A0201 (strong ligand), HISLHCYLV: A0207 (strong ligand), ISLHCYLVHL: A0201, ISLHCYLVHL: A0207, SLHCYLVHL: A0201 (strong ligand), SLHCYLVHL: A0207 (strong ligand), SLHCYLVHLL: A0201, SLHCYLVHLL: A0207
22	BCOR	Gly254Ser	BCOR_ENST00000342274_GLY254SER	LPPPHYVSPHIPSS	9.59	0	0.041	0.391	LPPPHYVSP: B5101 (strong ligand), PPPHYVSPHI: B5101, PPHYVSPHI: B5101 (strong ligand),
23	TCF15	Gly62ArgfsTer117	TCF15_ENST00000246080_GLY62ARGFSTER117	RRRRPPAALHLHLLPQQPAQGGWPS	4.58	4.22	0.044	0.386	PPAALHLHL: B5101, LHLLPQQPA: DRB1*0403 (strong ligand), LLPQQPAQG: DRB1*0803, LPQQPAQGGW: B5101 (strong ligand)
24	IRAK1	His85Arg	IRAK1_ENST00000369974_HIS85ARG	NRNARVADLVRILTHLQL	0	6.82	0.056	0.382	LVRILTHLQ: DRB1*0403 (strong ligand), VRILTHLQL: DRB1*0403, VRILTHLQL: DRB1*0803
25	KLHDC3	Leu90Phe	KLHDC3_ENST00000326974_LEU90PHE	YGHSTVLIDDTVFLWGGRND	14.3	0	0.027	0.379	TVLIDDTVFL: A0201, TVLIDDTVFL: A0207, VLIDDTVFL: A0201 (strong ligand), VLIDDTVFL: A0207 (strong ligand), VLIDDTVFLW: A0201, VLIDDTVFLW: A0207
26	ADAMTS14	Val266Ala	ADAMTS14_ENST00000373207_VAL266ALA	IEVLLAVDDSVVRFHGKEHVQ	13.06	1.68	0.025	0.373	VLLAVDDSV: A0201 (strong ligand), VLLAVDDSV: A0207 (strong ligand), VLLAVDDSVV: A0201 (strong ligand), VLLAVDDSVV: A0207 (strong ligand), VLLAVDDSVV: B5101
27	CYHR1	Ala47Val	CYHR1_ENST00000530374_ALA47VAL	AAGQAAAAALGEVAGPGLPDEAGLA	3.99	0	0.093	0.371	ALGEVAGPGL: A0201, ALGEVAGPGL: A0207
28	ZAN	Glu1262Ala	ZAN_ENST00000546292_GLU1262ALA	GASGRFVELQTAFGLRVRWDGDQQL	10.48	4.09	0.025	0.368	FVELQTAFG: DRB1*0403 (strong ligand), FVELQTAFG: DRB1*0803 (strong ligand), FVELQTAFGL: A0201, FVELQTAFGL: A0207, ELQTAFGLRV: A0201, ELQTAFGLRV: A0207, LQTAFGLRV: A0201, LQTAFGLRV: A0207
29	GDI1	Ser65Thr	GDI1_ENST00000447750_SER65THR	QLLEGPPETMGRGRDWN	7.11	0	0.051	0.366	QLLEGPPETM: A0201, QLLEGPPETM: A0207
30	CACNG8	Leu50Phe	CACNG8_ENST00000270458_LEU50PHE	YWLYTRAFICNTTN	10.93	2.18	0.027	0.350	YWLYTRAFI: DRB1*0803, WLYTRAFIC: A0201, WLYTRAFIC: A0207, WLYTRAFICN: A0201, WLYTRAFICN: A0207, YTRAFICNTT: A0201, YTRAFICNTT: A0207

Underlined optimized mutated sequence (Rank 23) indicates the frameshift mutation. AA Mutation: amino acid mutation, Optimized Mutated Sequence: long peptide neoepitope containing multiple short T-cell epitopes, Immunogenicity Score: the immunogenic potential of the encoded neoepitopes adjusted for homology with the Human proteome (Higher scores are indicative of a higher immunogenic potential.), VAF: variant allele frequency, Ancer^®^ Score: the parameter determined by EpiVax Therapeutics ranking the neoepitopes, which is based both on how mutations impact HLA- and TCR-interacting residues of predicted T-cell epitopes and on how high the immunogenic potential is.

## Discussion

### CTC collection using apheresis

The development and application of apheresis to isolate circulating tumor cells (CTCs) represents a significant advancement in cancer research and therapeutic strategies. Most studies utilizing apheresis for CTC collection have focused on evaluating treatment efficacy (19–21), prognostic predictions (22, 23), and liquid biopsies (24). The diagnostic leukapheresis significantly improved the detection frequency of CTCs, making it a clinically safe and effective method (25). Another study further emphasized the non-invasive nature of apheresis for collecting a substantial number of CTCs (26). While recent investigations have extended into RNA sequencing (27) and transplantation of cultured mammospheres into nude mice (28), our study is the first to successfully identify neoantigens using CTCs without nucleic acid amplification or extensive cell culture. The existence of malignant cells in CTC fractions was confirmed by cytological examination, and the lineage investigation by flow cytometric analysis revealed that at least 50% of cells in CTC fractions were not of hematopoietic origin, but were instead composed of cancer cells in relatively high purity. We could determine neoantigens from cancer patients without obtaining tumor tissue using cells sorted from the CTC fraction and normal blood cells. This success was enabled by the ability of our apheresis approach to yield a sufficient number of highly pure CTCs.

### Intratumoral and intertumoral heterogeneity

Cancer exhibits both intratumoral and intertumoral heterogeneity, which complicates the identification of neoantigens that reflect the tumor’s overall profile (29, 30). Studies have shown that only a small fraction of neoantigens is shared across multiple metastatic sites, with primary and metastatic lesions sharing an average of 19.6% of neoantigens in lung cancer cases (31). Another study revealed that only 4.4% of neoantigens were shared across all metastatic and primary lesions in a single patient (32). These findings underscore the necessity of incorporating information from multiple tumor sites to identify neoantigens that represent the entirety of the tumor’s genetic landscape. Previous study demonstrated that CTCs can monitor tumor heterogeneity and provide diagnostic value (11). In this context, CTCs offer a significant advantage by capturing genetic variations not detected in bulk analyses of primary tumors, and the ability of CTCs to provide comprehensive genomic information is particularly noteworthy. Several studies also indicate that CTCs may capture genetic variations not detected in bulk tumor analyses. For example, analyses of melanoma and breast cancer patients have demonstrated that driver mutations present in both primary and metastatic lesions can be detected in CTCs, as well as additional mutations from micrometastatic sites (33, 34). This evidence suggests that neoantigens identified using CTCs have the potential to represent the overall heterogeneity of the patient’s cancer, thereby enhancing the efficacy of personalized immunotherapy.

### Advantages of CTC-derived neoantigen identification

The identification of neoantigens using apheresis-derived circulating tumor cells (CTCs) provides several distinct advantages in the field of cancer immunotherapy. First, this approach enables non-invasive tumor sampling, eliminating the need for invasive biopsies of tumor tissues, which can be challenging and risky for patients. Second, it facilitates expedited initiation of personalized treatment strategies, as fresh cancer cells can be collected promptly for analysis and therapeutic planning. Third, the identified neoantigens have broad applicability across various therapeutic modalities, including peptide-based therapies, dendritic cell vaccines, and mRNA-based treatments, thereby expanding their potential utility. Lastly, neoantigen-based therapies have the potential to be combined with immune checkpoint inhibitors, enhancing their therapeutic efficacy. These features collectively establish CTC-derived neoantigen identification as a transformative tool in advancing the precision and effectiveness of cancer immunotherapy.

While promising, our study has several limitations. First, the extent to which CTCs reflect the genetic heterogeneity of all tumor lesions remains to be comprehensively validated. Although our findings align with existing evidence supporting the representational capacity of CTCs, further studies are required to confirm this observation across various cancer types. Second, it should be noted that CTC isolation by apheresis and FACS may introduce impurities and sampling bias, as leukocyte contamination and selection based on surface markers can affect both yield and clonality. Bulk sequencing may further mask intra-sample heterogeneity by averaging signals from mixed cell populations (35). Additionally, CTCs themselves are known to be highly heterogeneous, reflecting only a snapshot of dynamic clonal evolution and possibly underrepresenting minor subclones present in primary or metastatic sites (36). To address these issues, future work should leverage single−cell sequencing or employ multiple marker panels during isolation to more accurately capture CTC diversity and purity across cancer types. Third, the integration of neoantigen identification with other omics approaches, such as proteomics and transcriptomics, may provide additional insights into tumor biology and therapeutic targets. The EpiMatrix and JanusMatrix tools that are used in Ancer^®^ have been described in detail, previously (37). Fourth, optimizing the apheresis protocol to ensure the reproducibility and scalability of CTC collection is essential for widespread clinical application. Although this study did not assess the therapeutic utility of neoantigens identified from CTCs, our future plans include conducting clinical studies or clinical trials with larger cohorts to obtain clinical data and evaluate T cell responses against these neoantigens. We anticipate that such analyses will enable assessment of the immunogenicity of CTC-derived neoantigens. Furthermore, while this study did not compare neoantigens identified from CTCs with those derived from surgically resected tumor tissues, this comparison represents a critical next step. We intend to undertake this investigation in future research.

## Data Availability

The original contributions presented in the study are included in the article/supplementary material, further inquiries can be directed to the corresponding author.
